# Flexible Tactile Sensing Based on Piezoresistive Composites: A Review

**DOI:** 10.3390/s140305296

**Published:** 2014-03-14

**Authors:** Stefano Stassi, Valentina Cauda, Giancarlo Canavese, Candido Fabrizio Pirri

**Affiliations:** 1 Center for Space Human Robotics@PoliTo, Istituto Italiano di Tecnologia, Corso Trento, 21, 10129 Torino, Italy; E-Mails: valentina.cauda@iit.it (V.C.); giancarlo.canavese@iit.it (G.C.); 2 Department of Applied Science and Technology, Politecnico di Torino, Corso Duca degli Abruzzi 24, 10129 Torino, Italy; E-Mail: fabrizio.pirri@polito.it

**Keywords:** piezoresistivity, composite materials, percolation threshold, quantum tunnelling conduction, strain gauge, piezo-MEMS, flexible tactile sensor

## Abstract

The large expansion of the robotic field in the last decades has created a growing interest in the research and development of tactile sensing solutions for robot hand and body integration. Piezoresistive composites are one of the most widely employed materials for this purpose, combining simple and low cost preparation with high flexibility and conformability to surfaces, low power consumption, and the use of simple read-out electronics. This work provides a review on the different type of composite materials, classified according to the conduction mechanism and analyzing the physics behind it. In particular piezoresistors, strain gauges, percolative and quantum tunnelling devices are reviewed here, with a perspective overview on the most used filler types and polymeric matrices. A description of the state-of-the-art of the tactile sensor solutions from the point of view of the architecture, the design and the performance is also reviewed, with a perspective outlook on the main promising applications.

## 1. Introduction

Transferring the utilization of robots from the repetitive and limited tasks of the industrial environment to more complex operations for interacting with human beings has recently raised growing interest in both the research and applied technology fields. In this context, great improvements are required, not only for in-hand manipulation and exploration tasks, but also for safe operations and interactions with humans. Humanoid robots, unlike the industrial ones, are required to achieve their goals interacting with humans and their tools, adapting to the changes in the environment thanks to an autonomous learning process. In order to satisfy these requirements, robots need to be able to perform advanced human-like manipulation tasks such as rotation, translation and in-hand grasping [1–3].

To operate in changing environments, humanoid robots need to sense and elaborate the information about the surrounding environment, while interacting with real world objects. By analyzing the force and the position at all points of contact, robots can obtain information about the weight, the stiffness and the surface of a tool and elaborate a way to complete the assigned tasks. In order to satisfy these requirements, there is increased interest in the robotic community in the development of large area or whole-body tactile sensing structures. Without a high throughput tactile sensing system, humanoid projects strongly limit their interaction and cognitive capabilities [4]. Tactile sensing is also essential for fine manipulation tasks in humans. When our mechanoreceptors are anesthetized, like when our hands are chilled from cold weather, this results in a loss of sensing and our movements become inaccurate and clumsy. Simple operations like lacing up shoes or simply maintaining a stable grasp on an object can become very complex tasks. In order to reproduce human tactile sensing performances for fabricating sensor devices to be implemented in robot hands and bodies, several researchers have defined the guidelines and requirements which a robot tactile system has to satisfy for performing the basic in-hand manipulation tasks. These requirements, presented in Table 1, were determined by analyzing the human sense of touch, but even if they are almost exhaustive, they could be modified depending on the specific application in which the device would be used [3–7]. Moreover, even if some criteria are strict and technologically challenging, a possible solution to fulfill them could be complex systems integrating different devices instead of using a single tactile sensor.

In the last twenty years, many tactile sensor devices have been presented, exploiting several physical phenomena as transduction modes [2–4,8,9]. However, most of them do not satisfy completely the specific requirements of in-hand manipulation, being too bulky to be used without sacrificing dexterity or because they are fragile, rigid, slow or lack some fundamental characteristics. For this reason, it is not possible to choose a standard system like CCD or CMOS optical arrays used for the sense of sight. Moreover, tactile sensors get their information through physical interaction, this brings about problems of robustness to withstand several impacts and abrasions, and of compliance, to conform the device to the robot surface guaranteeing an adequate friction for handling tools securely [2].

The solutions presented in the literature for the fabrication of tactile sensors are innumerable, so that an in-depth classification based on task, site, transduction method and mechanical properties is necessary to organize and select the interested field [2–4]. The present review is concentrated mostly on the last two classifications, *i.e.*, transduction method and mechanical properties. Considering the mechanical properties, tactile sensors can be classified as rigid, flexible, compliant, conformable, stretchable, *etc.* Depending on the final application, the choice of these characteristics is fundamental for obtaining a perfect bonding and uniform coverage of the robot surface, and most of all for preventing damage and abrasion during the utilization.

The other classification is made with regard to the physical nature of the transduction method. Thus, tactile devices can be divided into piezoelectric [10,11], optical [12,13], magnetic [14,15], ultrasonic [16,17], resistive and capacitive [18–21]. With the first four solutions it is possible to obtain extremely high sensitivity and elevated spatial resolution, however most of these devices require a large pay load, are expensive and complex to fabricate, difficult to reproduce, and have reduced flexibility. Therefore they can result unsuitable for integration on a robot hand or body. In contrast, capacitive and resistive approaches guarantee wide working ranges, low cost and power consumption, and the use of simple read-out electronics. Most of them combine mechanical flexibility and resistance, providing a better integration and a primary protection from external overpressure, shock and vibrations. For these reasons, both capacitive and resistive approaches are certainly the most investigated among all the solutions. Moreover the majority of the commercial tactile sensors exploit these transduction mechanisms because of the lower cost and easiness of fabrication together with the basic electronics needed for the read-out operation. As major drawbacks, some capacitive and resistive solutions could lack of sensitivity and repeatability, mainly due to hysteresis phenomena or cross-talk between the sensor elements and thus their implementation could be limited in some high-precision application.

In these review we investigate the functional materials and the tactile sensor devices, presented in literature, exploiting flexible composites with piezoresistive properties. These materials are one of the best candidates to fabricate a sensing “skin”, able to reproduce the tactile sense and to fit the shape of the robot structure. Beyond the high conformability required to mimic the human skin, these composite sensing materials can be employed to generate devices with a wide range of sensitivity, a low power consumption and an elevate mechanical resistance guaranteeing protection from external physical agents that could damage the sensor. The major drawbacks of these types of devices are represented by the temperature sensitivity and hysteresis phenomena of the sensing response, which could influence the repeatability of the measurements [2,3].

We perform a classification on the basis of the piezoresistive conduction mechanism dividing the tactile sensors into piezoresistors, strain gauges, percolative and quantum tunnelling devices. For each flexible tactile device family we analyze the physics behind the conduction mechanism and we describe the state-of-the-art from the point of view of the material employed and the adopted architecture. The design and the performance are also reviewed, with a perspective outlook on the main promising applications. To introduce the following detailed analysis a general qualitative comparison of the four different tactile sensor types is presented in Table 2. Furthermore a table (Tables 3, 4, 5 and 6) with a quantitative comparison of each analyzed device is reported at the end of each section.

## 2. Piezoresistors

The work principle in piezoresistors consists in a variation of the resistivity of the material itself due to an applied stress. In general piezoresistors are made of silicon or other semiconductors, like germanium. Here the stress modifies the width of the band-gap and consequently the mobility of the charge carriers (electrons and holes). Therefore a significant variation of the resistivity is induced because of the dependence on mobility and density of the charge carriers [46]. Piezoresistors can also be fabricated using metals. Normally metals are mostly employed in the fabrication of strain gauges exploiting the resistance variation induced by changes in the geometry of the sensor, as explained in detail in the following section. However some metals, such nickel and platinum alloys, present a higher resistivity variation with respect to the resistance change induced by geometrical change. For these reasons many tactile sensors exploit metal piezoresistors either with simple geometry or as strain gauges, thus combining both resistivity and geometrical variation.

In order to minimize the size of the sensing element, MEMS technology can greatly contribute in exploiting the high piezoresistive responses of such piezoresistors. In this way several advantages can be further achieved, such as high sensitivity coupled with small size and ease of integration in MEMS devices and on printed circuit boards (PCBs), which can be used as sensor electrodes [47,48]. In general, resistive tactile sensors, as well as the capacitive ones, require typically a simple signal conditioning and therefore MEMS piezoresistors can provide a large number of sensing elements per unit area.

However, the major drawbacks of these semiconducting and metallic piezoresistors are their fragility and rigidity, together with the use of costly materials and temperature sensitivity. These disadvantages can be partly overcome by embedding the piezoresistors in flexible polymers, such as polyimide. From the material point of view, these solutions are not classical composites, where particles are homogeneously dispersed in a polymeric matrix. Here in contrast, most of the times rigid materials are integrated or embedded in a flexible substrate, in order to decrease the stiffness of the sensor, thus constituting a composite in a broader acceptation of this definition. Some examples were reported in the literature [49,50], showing the use of polyimide instead of fiberglass, which is commonly to make rigid PCBs. With a smart design on such flexible substrates, tactile sensors covering two-dimensional surfaces with small radii were demonstrated, even obtaining devices with the possibility of 3D sensing [51,52]. However, one must take into account that the increase of MEMS piezoresistor flexibility is at the expense of sensitivity and partly of miniaturization, with piezoresistive sensors sometimes becoming bulky.

**Figure 1.**
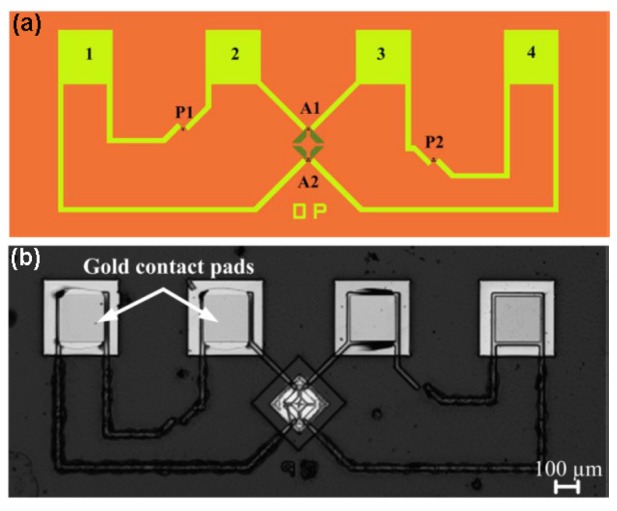
(**a**) Scheme of the MEMS force sensor showing the active (*A*1 and *A*2) and passive (*P*1 and *P*2) piezoresistors mounted in a half Wheatstone bridge configuration. (**b**) Optical microscope micrograph of the force sensor embedded in the flexible layer on top of the sensor © [2013]IEEE. Reprinted, with permission, from [22].

MEMS force sensors embedded in flexible polyimide substrates were developed by Ahmed *et al.* [22], showing the ability to monitor forces and pressures on nonplanar surfaces. A nichrome (Ni-80%/Cr-20%) film of about 35-nm in thickness was deposited by multiple lithographic patterning steps on flexible polyimide substrate and used to construct a half Wheatstone bridge geometry in a suspended aluminum oxide (Al_2_O_3_) membrane layer (Figure 1).

The half-Wheatstone bridge geometry was composed by two passive resistors *P*1 and *P*2 on the substrate and two active resistors *A*1 and *A*2 on the suspended membrane (see Figure 1a). In the ideal case all resistors are identical, *i.e.*, *P*1 = *A*1 = *P*2 = *A*2 = *R*, and the change of the active resistances with strain are also equal, *i.e.*, *ΔA*1 = *ΔA*2 = *ΔR*. Thus, the change in output voltage Δ*V_out_* of the bridge biased with *V_in_* is:

(1) $$\Delta V_{\text{out}}=\frac{\frac{\Delta R}{R}}{2+\frac{\Delta R}{R}}V_{in}\approx \frac{\Delta R}{2R}V_{in}$$

Therefore, the normalized change in resistance *ΔR*/*R* can be calculated from the change in output voltage *ΔV_out_* for a given input voltage *V_in_*. The measurement of the MEMS force sensor was carried out assuming the change in output voltage between a no-load and full load conditions and using a load cell.

An almost linear curve of the *ΔV_out_* for a given input voltage *V_in_* was obtained by gradually increasing the applied compressive force. The maximum applied force was about 2.5 mN over an area of 283 μm × 283 μm for the sensor device, obtaining a force sensitivity of the sensors between 0.266 V/N and 2.248 V/N, with an average sensitivity of 1.25 V/N. The average gauge factor, calculated by the authors combining the response measurements and the simulation of the designed sensor, was 1.75.

Despite the high piezoresistive response, piezoresistor materials made of silicon or metals such as NiCr or Cu-Ni on flexible substrates, as those mentioned above, suffer of ease of fracture under a large applied load. Therefore polymer-based composites filled with conductive particles are preferable for their broader range of application. In particular Carbon NanoTubes (CNTs)-polymer composites were already broadly used as mechanical strain sensors, since their resistance changes upon an applied pressure.

**Figure 2.**
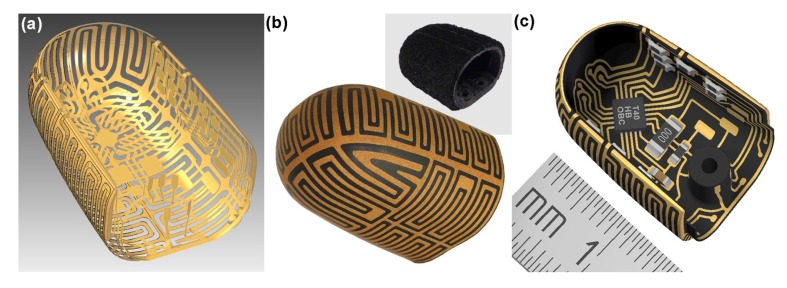
(**a**) The CAD image of the 3D-shaped electrode tracks; (**b**) The fingertip obtained after LDS process and chemical baths, with the integrated sensing conductive foam. Inset: the milled 3D-shaped sensing foam with mounting bracket; (**c**) The finished tactile sensor with embedded data acquisition electronics on the backside of the sensor © [2013] IEEE. Reprinted, with permission, from [23].

Koiva *et al.* [23] developed a compact tactile sensor fingertip with embedded electronics using a Laser-Direct-Structuring (LDS) process (Figure 2). Thanks to this structuring process, the tactile sensor was designed to be added to free-form PCB surfaces with the remarkable possibility of creating very fine structures, down to 100 μm. The resistive sensing working principle was achieved using conductive metal tracks as electrodes and conductive foam or rubber as the sensor material. The possible sensor materials can also be composites, such as elastomer foam or rubber with added carbon particles or conductive fabrics. Therefore the coupling between the flex-printed PCB and the elastomeric sensing material allowed the authors to produce a tactile sensor deformable up to a small radius and with almost arbitrary 3D free-form shapes. The signal digitalization was obtained by converting into a voltage the resistance measured between the two electrodes, or an electrode and a common ground-plane shared by all tactels of the sensor array. In particular, a simple and constant pull-up resistor was attached to a constant power supply (voltage divider circuit). The voltage at the junction of the resistors was sampled by an analog-to-digital converter (ADC), therefore the data were provided in a digital form for either transmission and further signal processing. The measurement range could be easily shifted by varying the pull-up resistor value. In particular, higher resistances allowed the measurement of lower applied pressures, however higher signal-to-noise was detected and the maximum measurable applied strain was limited. To reduce the signal-to-noise ratio, the author directly integrated both the circuitry for analog voltage measurements and the digital communication into the fingertip. In particular as ADC, they developed a programmable module in the fingertip so that the data acquisition was managed in terms of high protocol configurability thus leading to good adaptability to different hardware systems. Using an internal 8 MHz clock, the chip required only one capacitor and a resistor (as external components) to operate. It featured 12 ADC inputs with a sampling resolution of 10 bits and a maximum combined sampling frequency of 40 kHz. The fingertip tactile sensor was finally equipped with 12 tactels, resulting in an average spatial resolution of about 5.5 mm. The performances of the sensing fingertip were evaluated by a customized measurement bench capable of exerting forces from 0 N to 80 N. The authors showed that their tactile sensor was quite sensitive to first touch (detection of 0.03 N/cm^2^) and the signal repeatability was very high. In addition, slip detection was also possible, since high sampling rates, at around 1 kHz, were used.

**Figure 3.**
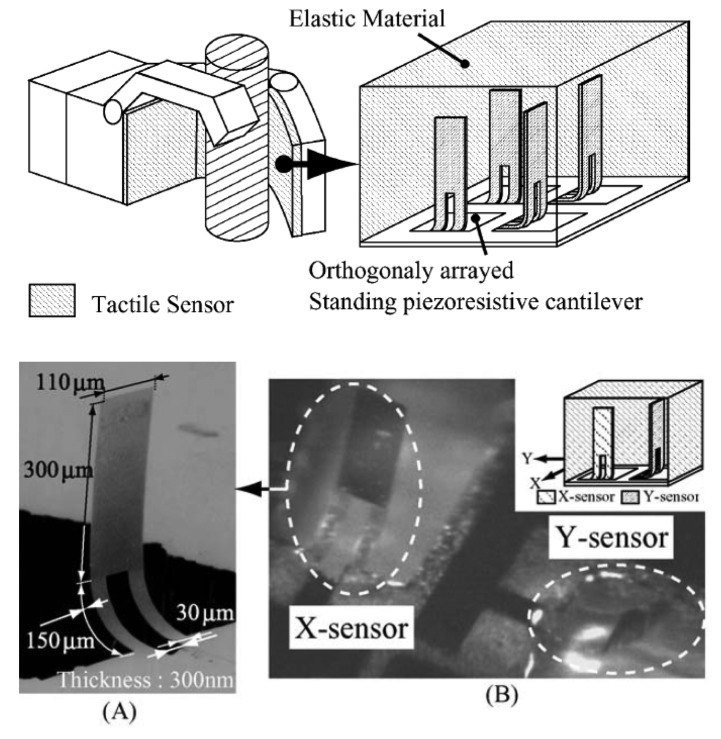
(**Top**) Schematic of the tactile sensor fabricated with standing cantilever with piezoresistorfor shear stress detection. (**Bottom**) (A) FESEM and (B) photo of the standing cantilevers in PDMS. Reprint from [24], Copyright (2006), with permission from Elsevier.

Noda *et al.* [24] proposed and fabricated a tactile sensor based on silicon piezoresistors able to independently detect the shear stress in the two axial components. It consisted of an array of vertical piezoresistive cantilevers standing orthogonally to each other, thus able to detect the directions and the magnitudes of the applied shear stresses. The cantilevers were prepared by microfabrication technology from a silicon on insulator (SOI) wafer. The horizontal cantilevers were then vertically aligned with the help of a magnetic field which interacts with a Ni magnetic layer previously deposited on the SOI surface. To maintain the cantilevers standing without the magnetic field, parylene-C was vaporized on their surface. Finally the whole cantilever array was embedded in an elastic and flexible PDMS support and tested under shear stresses in the X- and Y-directions (Figure 3). In this way, the standing cantilevers follow the elastic deformation of the PDMS material. The authors successfully reported the detection of a Δ*R*/*R* resistance variation for the −5.0 to 5.0 kPa shear stresses applied in vertical direction to the cantilever. The measured sensitivity was 20 times higher than that obtained for the shear stress applied in parallel direction. This result showed that the sensor was able to detect and distinguish one axial component of applied shear stress, with a 10% error occurred in the magnitude measurement of the shear stresses. The authors also proposed, as a future improvement on the sensor's sensitivity, to change the elastic material.

In another paper [25], a flexible, a soft and compliant tactile microsensor (SCTM) also based on a silicon piezoresitor was reported. The 3-D silicon microsensor was 1.4 mm^3^ in size and was embedded in a flexible and robust packaging having a minimum thickness of 2 mm in order to be finally integrated in an anthropomorphic artificial hand. With this aim, tests of static calibration, maximum load, noise and dynamic characterizations were accurately carried out on the SCTM together with the electronic hardware and a processing algorithm. It was demonstrated that the SCTM had a higher loading range with respect to the bare silicon microsensor (only about 3 and 0.5 N for normal and shear loads, respectively). Indeed the integration of the sensor into a soft and flexible polyurethane material allowed the tactile sensor to withstand forces even higher (about 15 and 11 N for the normal and tangential static loads, respectively) to those involved in human fine grasping activities (about 4 N). In addition the authors focused on slippage experiments, demonstrating that their SCTM was sensitive enough to detect a slip event, using contact surfaces with different roughness (aluminum and sandpaper probes). They also developed a customized processing algorithm in order to detect the instant of a slip event.

## 3. Strain Gauges

Strain gauges are electrical conductors whose resistance depends on their geometry. When the conductor is stretched at a level below its breakage or its permanent deformation, it becomes longer and its cross section thinner, thus inducing an increase of its electrical resistance. Normally strain gauges are designed as long and thin conductive patterns, arranged in a zig-zag configuration of parallel lines. In this way, a tensile stress in the direction parallel to the array of conductive lines results in a stretching of each line at the same time and in a sum of the resistance increase of each conductive path.

In general the gauge factor of a thin metallic pattern is between 2 and 5 [53], thanks to the changes in either the length or the cross-sectional area:

(2) $$GF=\frac{\Delta R/R_{0}}{\varepsilon }$$

When assuming constant both the resistivity and the volume of a metal film with resistance *R*, stretched to length *L*, having *R_0_* and *L_0_* as the corresponding initial values and mechanical strain *ε* [31,54]:

(3) $$\frac{R}{R_{0}}={\left(\frac{L}{L_{0}}\right)}^{2}$$

since:

(4) $$\frac{R}{R_{0}}=1+\left(\frac{\Delta R}{R_{0}}\right)$$

and:

(5) $$\frac{L}{L_{0}}=1+\varepsilon$$

when *ε* < < 1:

(6) $$\frac{\Delta R}{R_{0}}\approx 2\varepsilon$$

which corresponds to a gauge factor of 2.

Strain gauges can be also made of semiconductor components, which exhibit a larger gauge factor if compared to metallic foils, due to the combination with the previously described piezoresistive effects. In this case, the resistivity changes quickly with the applied strain due to the dependence of the bandgap on interatomic distance, while the variations induced by geometrical change are smaller or, in the case of very simple geometry, almost negligible. It was indeed reported that the gauge factor of MEMS based on p-type single crystalline silicon can reach a value of 200 [55].

The advantage of having MEMS strain gauges is based on the integration in a reduced space of a high-density sensor array and connection to electronics for signal processing. In particular, some efforts have been devoted in developing bio-inspired MEMS-based strain gauges sensors, thus able to mimic the form and functions of biological sensing systems, *i.e.*, as smart sensing skins. However the limited data gathering capabilities, the low deformability, high fragility of strain gauges, and general packaging difficulties have limited the use of such MEMS-based sensors in tactile devices.

To overcome the fragility issue, several authors have reported the embedding of strain gauges in polymeric flexible substrates [56,57] or covering the strain gauges with a protective polymeric layer [58]. The first attempts indeed were based on composite strain gauge structures, based on the integration of rigid elements, such as miniaturized metallic serpentines on polymeric substrates, able to adapt their geometry to the applied deformations, requested in the tactile sensing application field. However, such composed structures have not proven to be a reliable interface between a robotic manipulator and the manipulated object.

**Figure 4.**
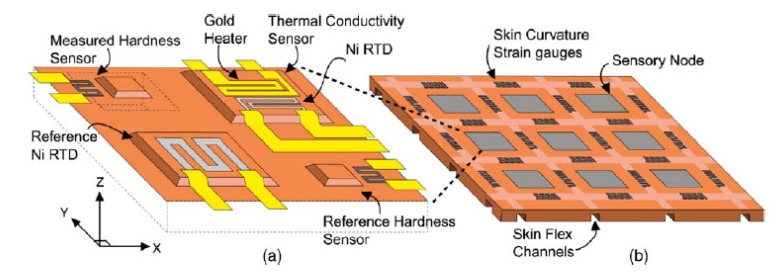
Scheme of the multifunctional sensing skin. (**a**) The single sensor node showing the four incorporated sensors: the reference temperature sensor; the thermal conductivity sensor; the contact force and the hardness sensors. (**b**) Each sensor node is arranged in an array to form the sensing skin, with skin curvature strain gauges, to map the skin surface. Reprint from [26], Copyright (2004), with permission from Elsevier.

Engel *et al.* [26] developed a multifunctional polymer-based sensing skin, trying to mimic some design and functionalities of the human skin. The developed device was able to sense the hardness, the thermal conductivity, the temperature, and the surface profile of an object. It was constituted by an array of sensor nodes, each composed by four distinct sensors exploiting the different functions (Figure 4). For the temperature measurement and compensation, a reference nickel Resistance Temperature Device (RTD) was employed. The thermal conductivity was measured with a gold heater and nickel RTD pair, and a membrane with a strain-gauge based on nickel-chrome alloy (NiCr, 80:20 wt.%) was used for contact force and hardness sensing. Finally a NiCr strain gauge was employed as reference contact force and hardness sensor. The multimodal sensor skin was then built on a flexible polyimide substrate.

The authors measured the hardness by a differential measurement among two resistances in the range of 10 to 80 Shore A. The resistance variation of each sensor was converted to a displacement measurement using calibration data, obtained by the change in resistance of the measurement sensor and the reference sensor in response to a known normal displacement. This trailblazing study however showed several limitations due to wires connections. Indeed the use of the sensor in a real application is limited by the scalability of the wiring interconnects. Indeed, each node requires about 10 wires, and since they were closely packed (see Figure 4) with only a 5 × 5 array of sensing nodes covering an area 25 mm × 25 mm, about 250 wires were required to collect all the data. It is therefore necessary a distributed signal processing and multiplexing architecture in order to integrate this structure on a wide area robotic sensing skin.

**Figure 5.**
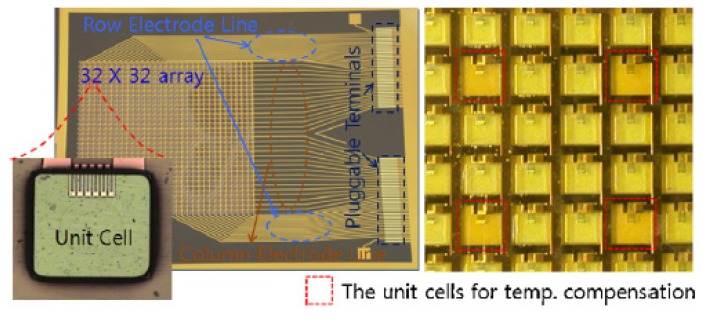
The NiCr strain gauge sensor array (32 × 32) with interconnection terminals. The inset displays a unit tactile sensor cell in the sensor array. Dashed red lines show the unit cells for temperature compensation in the sensing array. Reprinted from [27], Copyright (2009), with permission from Elsevier.

In another study Kim *et al.* [27] fabricated a polymeric MEMS flexible tactile sensor array together with interconnection terminals on the same polymer substrate, in order to integrate them as a sensor module (Figure 5).

The tactile sensing arrays were made as 4 × 4, 8 × 8, 16 × 16 and 32 × 32 sensing elements, constituted by NiCr strain gauges, and a flexible flat cable for signal interconnection. The size of the sensor unit cell is 1 mm × 1 mm and the overall sensing module size is 5.5 cm × 6.5 cm. To measure the sensor characteristics through the integrated interconnection terminals, normal force ranging from 0 to 1 N was applied to a tactile sensor unit. The measured resistance increased linearly with the normal force in the range of 0–0.6 N, however a decrease of the resistance rate variation was observed above 0.6 N.

A microscale biomimetic tactile sensor with epidermal ridges was proposed by Zang *et al.* [28] to enhance the sensitivity of force detection. In particular the authors fabricated artificial epidermal ridges made of polydimethylsiloxane (PDMS), with 400 μm in width and 110 μm in height, and placed them on micro-fabricated copper strain gauge arrays, deposited on a polyimide substrate. The authors measured an increase of about 1.8-fold in the sensitivity of the strain gauge thanks to the artificial epidermal ridges, with respect of the same sensor without ridges. However this study lacks in the electronic design, sensor integration and in the evaluation of the specific sensor figures of merit.

In a similar approach, Choi *et al.* [29] fabricated nichrome strain gauges on a polyimide film using a polymer micromachining technology. This flexible and three-axial tactile sensor can detect normal and shear loads and can be applied in a curved or compliant surface that requires slip detection and flexibility, such as a robotic fingertip. In particular the authors evaluated the optimal positions of strain gauges through strain distribution from finite element analysis. The sensor was experimentally tested by applying normal and shear loads from 0 N to 0.8 N and leading to sensitivity values of about 206.6 mV/N for normal load and 70.1 mV/N for shear stresses. The composite structure showed a force capacity of 0.6 N in the three-axis direction and good linearity.

To solve the wiring problems discussed above, Tata *et al.* [30] proposed a wearable strain gauge tactile sensors with wireless connections and designed for human motion detection. A 0.5 μm thick amorphous carbon was deposited by sputtering on a 125 μm thick polyimide film and laser micromachining was applied to design the strain gauge pattern. Finally PDMS was used to package the whole sensor, having final dimensions of 0.5 mm in width and 10 mm in length. The wearable unit was also equipped with an interface circuit able to convert the sensor analog signals into digital format feeding to a microprocessor. The signals were then sent wirelessly to a reader module, and the data were displayed on a remote computer, while being recorded continuously. The sensor was calibrated and the entire sensor and wireless system were mounted as a unit on a knee joint and tested during various types of physical exercises. This device presented several versatilities in the construction system and integration with electronics that can be easily exported to robotic tactile sensing applications.

**Figure 6.**
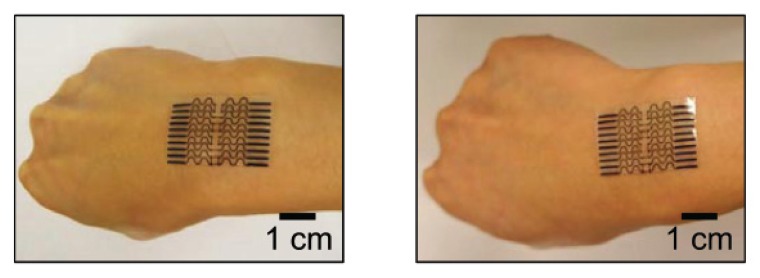
The all-elastomer composite strain gauge with a flexible and compliant structure. The images show the device mounted on the wrist at different levels of bending. Reproduced with permission [31].

Lu *et al.* [31] similarly reported on a tactile sensor conceived for the measurement of physiological parameters of the human skins. However, it is an interesting example of tactile sensor which technology can be easily applied not only to the monitoring of biomedical parameters, but also to robotic sensing skin application. In particular the authors fabricated an all-elastomer strain gauge system, showing a high gauge factor (about 29) and flexibility, having a Young's modulus of about 244 kPa, thus falling in the range of the human skin. The device is made of composite carbon black (CB)-PDMS resistors for the fabrication of strain gauges, because of their high resistivity and strong dependence on the applied strain. In addition a thick, carbon nanotubes (CNT)-PDMS composite conductors are used for the interconnections, due to their relatively low resistivity and weak dependence on strain. An insulating PDMS matrix is also used as substrate (Figure 6).

The overall device had an electrical response that depended almost entirely on the strain in the CB-PDMS and can be laminated on, forming a conformal contact to the human skin. The measured strains of the device when applied to human skin were between 11.2% and 22.6%.

## 4. Percolation Mechanism

Functional materials composed by an insulating matrix and conductive filler were widely studied because of the tunability of the pressure sensing range varying composition and materials. The motion of the particles inside the matrix, generated by the applied load, induces a rearrangement of the conduction path and thus a variation of electrical resistance. Depending on the kind and shape of the filler, the conduction mechanism inside the composite could be percolation or quantum tunnelling (or also a combination of both) and the applied load could cause a decrease or an increase of the conductivity.

In general a decrease of the electrical resistance is registered with low aspect ratio particles, such as metal powders and carbon black [59–64]. This effect is called the negative pressure coefficient of resistance (NPCR) effect. In contrast the resistance increases with compressive strain with high aspect ratio particles, *i.e.*, carbon nanotubes (CNTs), graphite nanosheets and high structure carbon black agglomerates [32,65–71], leading to the positive pressure coefficient of resistance (PPCR) effect. The behaviour of these latter materials is described by the percolation theory, where the prompt insulator-conductor transition occurs in correspondence of a small variation of the conductive filler fraction defined as percolation threshold [72,73].

In contrast, in the NPCR group, two conduction mechanisms could occur, *i.e.*, percolation, as in the PPCR case, and quantum tunnelling. The second mechanism will be described in detail in the following section. Anyway, one has to keep in mind that the composite is normally described by the predominant conduction mechanism, but also other conduction events attributable to different mechanisms are present, even if they are normally negligible [74].

All the composite materials made up of a polymeric matrix and conductive fillers behave like an electrical insulators for concentrations of particles below the percolation threshold. Upon rising the filler concentration, the gap between two neighboring particles decreases to bring them in contact, thus leading to the formation of a local conductive path. When the local conductive paths are enough to cross the whole composite thickness, an effective conductive path is formed, producing a sharp increase of the bulk electrical conductivity of the material [75].

A simplified description of the percolation conduction mechanism can be provided by considering the material as a porous medium and the electrical current as a liquid flowing through this medium. Then one has to evaluate the probability of the liquid passing through the porous matrix and reaching the base. The composite is designed as a three dimensional matrix composed of points and connections that could be opened and closed, controlling the flow of the fluid. Any connection has a probability *p* of being open, letting the fluid flow, and *vice versa* a (1−*p*) probability of being closed. Considering the matrix as an infinite network, a probability of the fluid crossing the whole material also has to be defined. This global probability has two limit conditions (0 corresponding to electric insulator behaviour and 1 to electric conductor) and is a function of the local probability. Therefore there is a critical probability *p_c_* to switch the global probability of the system from 0 to 1, creating a path for the fluid to flow to the base. Similarly a critical concentration *x_c_* is defined around which there is a large conductivity (*σ*) variation with small concentration variations, switching the system from an insulating to a conducting behaviour:

(7) $$\sigma \propto {\left(x-x_{c}\right)}^{t}$$

where *t* is the critical exponent that determines the trend of the function around the critical concentration [76]. Normally the percolation threshold is inversely proportional to the aspect ratio of the particles.

A similar process could take place under the application of a compressive force. Without any load the particles are distant enough to guarantee an insulating behaviour, while when the composite is deformed, the particles come closer, touching each other, creating conductive paths that decrease the electrical resistance of the sample [75]. Actually this process is valid mainly for low aspect ratio particles, which tend to have a spherical shape. In fact in this case the compression could only induce a reduction of the polymeric interparticle gaps and thus an approaching between closer particles. For high aspect ratios, the applied force could cause also a reorientation and reshaping of the particles that could induce the destruction of the existing conductive network or a reduction of the conductivity of the filler [70,77].

CNTs with different aspect ratios [32,66–69], graphene [70] and high structure carbon black [65] were for example used in different silicone-based nanocomposites, either methylvinyl silicone rubber (VMQ) or polydimethylsiloxane (PDMS). These composites are generally prepared by dispersing the carbon-based filler in the polymeric matrix by wet mixing conditions, thus employing a solvent and an ultrasonicator to guarantee a good dispersion [68]. After the solvent evaporation, the composite materials are cured and cut or moulded in different shapes for further testing. Electrical contact is afforded by metal sputtering or conductive paste obtaining full coverage of the top and bottom surface or patterned electrodes.

**Figure 7.**
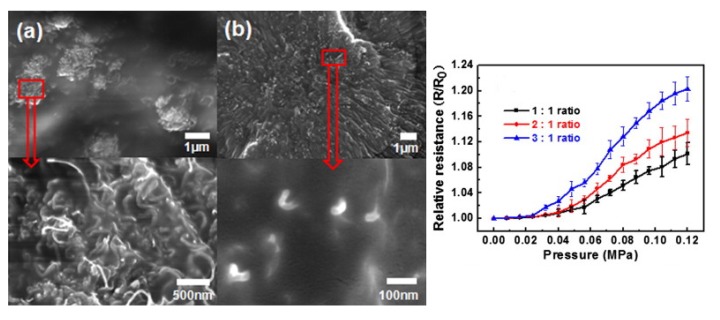
(**Left**) SEM images of fractured surfaces of MWCNT/PDMS composite (a) without and (b) with thiopene molecules (1:1 weight ratio respect to CNT), showing the improvement in the dispersion process of the carbon nanotubes (white fibers in the images). (**Right**) Piezoresistance variation of the composite material as function of the thiophene molecule-MWCNT weight ratio. Reprinted from [32], Copyright (2011), with permission from Elsevier.

Considering the CNTs, their extremely high aspect ratio ensures the formation of a conductive network in the nanocomposite at very low CNTs content. With respect to carbon black or metallic fillers, showing a much lower aspect ratio, the lower amount of CNT required to reach the percolation threshold results in better mechanical properties of the final polymeric composite, low viscosity and storage modulus, and finally less product costs [78]. Despite their advantages, CNTs can easily aggregate and form entanglements between them due to the intermolecular van der Waals forces. It is therefore difficult to homogeneously disperse CNTs in a polymer matrix; such a disadvantage has prevented the broad use of CNTs in piezoresistive composites. Many groups have tried to solve the problem by functionalizing the surface of CNTs with methyl (-CH_3_) or amine (-NH_2_) groups [66,79]. However the surface modification of CNTs can degrade both their electrical and mechanical properties, as well as cause shortening of the CNTs, thus reducing the advantages of having high aspect ratio conductive fillers. Hwang *et al.* [32] have reported on a successful functionalization of multi-walled CNTs (MWCNT) by conductive thiophene molecules, thus obtaining well-dispersed and non-entangled CNTs fillers in a PDMS matrix and still guaranteeing low particle content. The overall resistance of the composite rises by increasing the external pressure, leading to the PPCR effect. The composite shows a piezoresistance response in the small pressure range 0–0.12 MPa, strongly dependent on the concentration of the thiophene molecules, as shown in Figure 7.

Recently, Pyo *et al.* [33] have proposed a flexible tactile sensor based on four CNT-PDMS sensing parts placed on a polyimide substrate with Cr/Au interdigitated electrodes and surmounted by a bump structure made of SU-8. In details, a 2% in weight of CNTs were dispersed into the PDMS copolymer by further addition of curing agent at a ratio 1:10 with respect to the CNT-PDMS mixture. The final dimensions of the sensing composite part were 3 mm × 3 mm × 20 μm. When a compressive uniaxial load was applied on the bump, the four composite parts were uniformly and equally compressed and the overall electrical resistance increased. In addition, when a shear force was applied, the four piezoresistive composites responded in different manners, therefore the contribution of this lateral force could be easily discriminated from a uniaxial compressive one. The authors measured the linear variation of the relative resistance as a function of the normal force, applied with a micromanipulator from 0 to 2 N, as measured by a load cell. They obtained a *ΔR*/*R* varying from 0, at zero applied force, to about 14% at 2 N.

**Figure 8.**
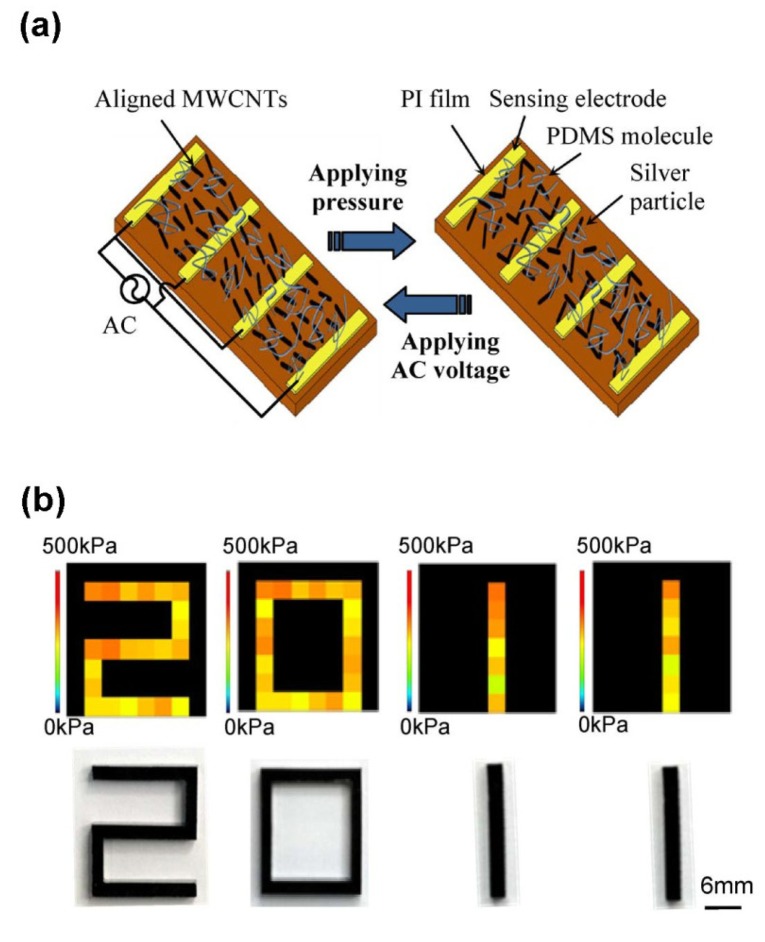
(**a**) Scheme of the tactile sensor working principle, where the CNTs are aligned through DEP across the interdigitated electrodes (left panel); After a compressive stress, the CNT conductive path is destroyed, increasing the resistivity of the overall composite material. (**b**) The PMMA stamps (bottom panel) used to apply a pressure on the tactile sensing composite. On the top part of the figure, the shapes of the stamps are retained from the sensor, as visualized by a software interface © [2012]IEEE. Reprinted, with permission, from [34].

In another interesting work, Lay *et al.* produced a tactile composite sensor by aligning CNTs into a PDMS matrix through dielectrophoresis (DEP) on interdigitated electrodes [34]. The novelty of the presented approach is that the array of CNT was capable of retaining over time the resistance variation upon an exerted uniaxial pressure. At the beginning, the aligned CNTs formed a conductive path among the interdigitated electrodes. When compressing the composite with an external force, the array was distorted and the conductive paths were removed, thus leading to an increase of the overall resistivity of the composite material. This effect could be however easily erased, bringing back the composite resistivity to the initial value, by applying again the DEP process among the interdigitated electrodes (Figure 8a). By adding conducting silver nanoparticles together with the CNTs, the resistivity of the CNT-based composite was further lowered, thus matching the dynamic range of the sensor read-out circuit. In particular the authors integrated the resistivity read-out circuit with the DEP driving source. Two multiplexers were employed to provide the driving voltage to the composite sensor and to receive, by signal scanning, the acquired signals from it upon pressure application. The sensing data were then sent to a PC for visual representation. To display the effectiveness of their approach, the 8 × 8 sensor matrix was compressed by different solid PMMA shapes, retaining over time the shape of the used stamps when the applied force was removed (Figure 8b). The applied shapes can be therefore visualized by a software interface. By re-applying the DEP signal, the images of the stamps could be erased.

**Figure 9.**
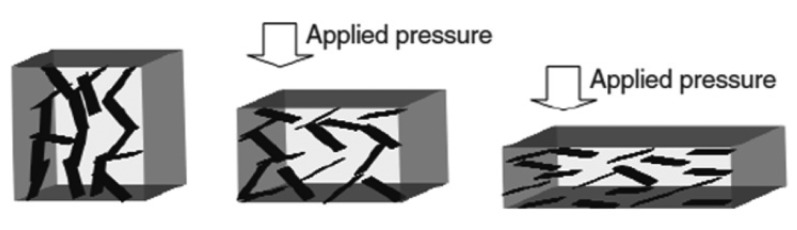
A schematic of the mechanical behaviour of graphene/graphite composite under the application of a uniaxial force. Reproduced with permission [71].

The PPCR effect was also verified in piezoresistive nanocomposites using graphene or graphite as conductive fillers [70]. In particular it was reported that these graphene-PDMS composites can reach high levels of sensitivity (*R*/*R_0_* > 400 under the pressure of 1.2 MPa) with a very small graphene content (only 1.19% vol), as well as excellent repeatability, small hysteresis, long-term durability and soft and flexible composite materials. The authors referred the exponential increase or the *R*/*R_0_* with the applied pressure due to the high specific surface area of graphene, all forming a conductive network at low particle content. Indeed the graphene nanosheets in such low amount in the matrix were able to reorient along the in-plane direction during the uniaxial compression and follow the movement of the polymeric chains. This led to the continuous destruction and reconstruction of the conducting network, resulting in an overall decrease of direct contacts between the nanosheets and a dramatic increase of the average electron tunnelling distance, as shown in Figure 9.

**Figure 10.**
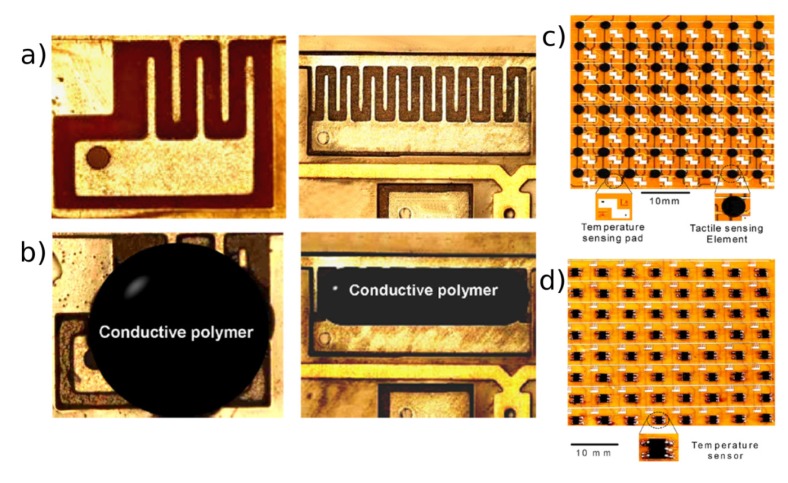
(**Left**) Two different pattern of interdigitated electrodes used by Yang *et al.* in their tactile sensor, (**a**) before and (**b**) after the deposition of the piezoresistive composite. (**Right**) Image of (**c**) the top of the sensor containing the tactile sensing elements and the temperature sensing pads, and of (**d**) the back with the temperature sensors. Reprinted from [36], Copyright (2007), with permission from Elsevier.

The majority of tactile sensor devices exploiting the percolation mechanism employed composite materials containing carbon black as filler in a volume percentage close to the percolation threshold, thus showing NPCR effect [35,36,40,80]. The classic design of these tactile sensors is based on discrete sensing cells disposed in a homogenous array to detect the applied load. The measurement is performed by applying a voltage and reading the resulting current from each tactel, by means of a multiplexer, until all the matrix is covered. The tactels can mount two different electrode configurations [81]: double sided contact, by crossing horizontally and vertically aligned electrode lines on the two sides of the piezoresistive composite [40,42,82], and single sided contact, by placing both the electrodes on the back side of the sensing material, normally creating an interdigitated pattern. Flexible tactile sensors implementing interdigitated electrodes with a carbon piezoresistive composite were presented by Yang *et al.* [35,36]. In their work an array up to 32 ×. 32 sensing element on copper metallized polyimide (PI) film was fabricated defining the electrodes by a micromachining technique and then spotting the composite material using a numerical-control dispenser, as shown in Figure 10. Moreover the same number of temperature sensing chips were integrated on the back of the PI film and employed as temperature-sensing cell, still preserving the flexibility of the whole device. By means of a dedicated scanning circuit and acquisition and visualization software, they were able to obtain simultaneously a map of the temperature and applied load on the sensor.

Another multifunctional tactile sensor device was proposed by Yuji *et al.* [37]. This solution is based on a single composite material, able to sense not only the contact force, but also the temperature change and the contact face number per one tactile sensor. This interesting goal was achieved by using four pressure-conductive rubber sensors electrically connected in parallel and a selective data processing method. When a pressure is applied to one of the four material faces, the contact resistance of the relative rubber face decreases. In addition, for equal applied pressures, the resistance values rise by increasing the temperature in a range from 20 to 40 °C and this temperature dependence is obtained in the first 1 to 5 s. Thus this time can be used to acquire the temperature values from the sensor device, then after 5 s the resistance variation can be used to monitor the pressure value and the contact face number. Based on this time parameter, the authors then developed an architecture for data processing able to select, depending on the input time, the input values and to discriminate their contribution, obtaining multifunctional data processing and information relative to the temperature, pressure and contact face number.

**Figure 11.**
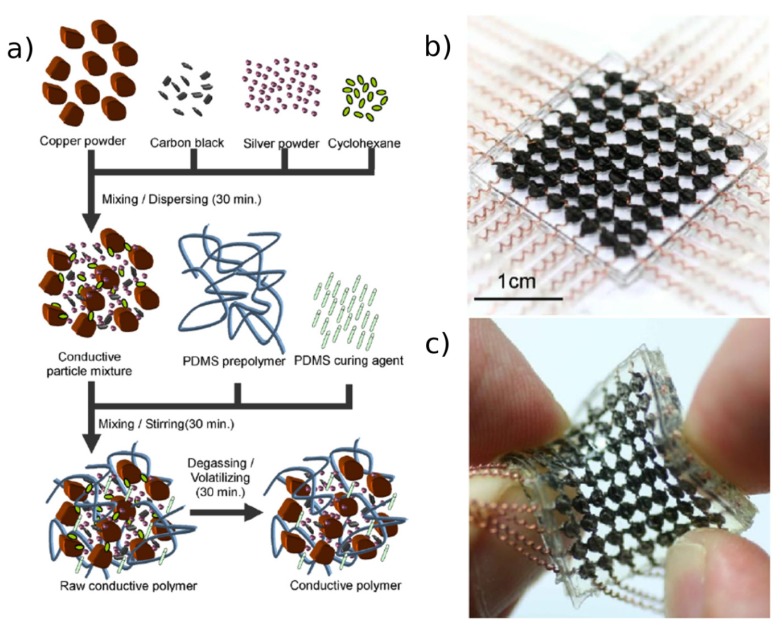
(**a**) Process flow of the preparation of the piezoresistive composite. (**b**) Image of the whole sensor showing each tactels composed by the composite disposed at the intersection between the spiral electrodes. (**c**) Example of the high stretchability and twistability of the sensor. Reprinted from [39], Copyright (2009), with permission from Elsevier.

A novel architecture was proposed by Cheng *et al.* to fabricate an anthropomorphic robot skin with a large 8 × 8 area of highly stretchable tactile sensing arrays [38,39]. PDMS was employed both for the fabrication of the skin structure and as matrix of the piezoresistive conductive composite with carbon black, copper and silver powder as fillers (Figure 11a). The sensing material was placed in the spacing of orthogonally crossing spiral wire electrodes that can withstand high deformation and twisting up to 70° without any damage in the structure or functionality, as shown in Figure 11c. The electrodes were prepared by rolling copper wires around nylon line by means of a winding machine. The array was integrated in a flexible PDMS film, shaped as an arm, and mounted and tested as sensing skin. Integration of wires into a piezoresistive composite was also exploited by Shimojo *et al.* mounting four different sensing arrays, with a high sensitivity in the range 0–200 kPa, on the tip of each finger of a robot hand [40]. The sensors were tested in grasping simple shapes (cone, sphere, column and human arm) and they were able to successfully characterize all the steps of the operations and distinguish between the different forms.

**Figure 12.**
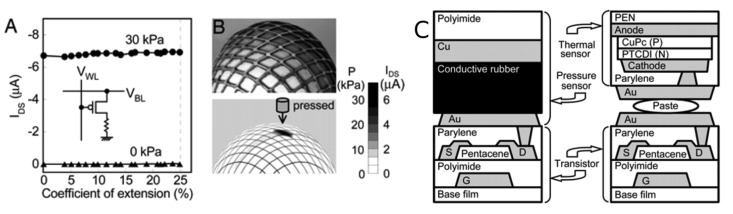
(**A**) Source-drain current as function of the expansion of the device with and without application of pressure. (**B**) Image of pressure sensor matrix put on an egg and the corresponding spatial distribution of pressure under the application of local load. (**C**) A schematic of the structure of the pressure and the thermal sensor cells with the organic transistors. Reproduced from [41], Copyright 2005 National Academy of Sciences, USA.

Another interesting design is the coupling of the piezoresistive composites with flexible organic field effect transistors (OFETs) that integrate a sensor element with the acquisition electronics [41,83,84]. Somaya *et al.* fabricated an OFET on a polyimide or a poly(ethylenenaphthalate) film, depositing pentacene as channel layer and polyimide as gate dielectric layer. The transistors show a mobility of 1 cm^2^/Vs in the saturation regime and an on/off ratio around 10^5^–10^6^, sufficient values to obtain a well-defined mapping of pressure. The top of the transistor is then coupled with a layer of piezoresistive composite between two electrodes, one of them connected to the drain of the OFET, as shown in Figure 12c. In this configuration of a fixed gate voltage, when the resistance of the composite decreases, the source-drain current increases proportionally. This configuration guarantees a high sensitivity (10 kPa of minimum detectable pressure) and a much lower power consumption compared to the classical device which simply measured the electrical resistance. Moreover the sensor was completely stretchable and deformable, showing a stable signal expanding the device up to 25% of the initial dimension, with and without the application of a pressure, as shown in Figure 12a. Additionally, the pressure sensor array was integrated with an array of thermal sensors in order to monitor the two physical quantities simultaneously. The thermal sensors were manufactured by coupling on the top of the OFET an organic diode and connecting it in series to the drain [41].

## 5. Quantum Tunnelling Conduction Mechanism

As already reported above, the piezoresistive working principle coupled with flexible composite materials can fulfill several requirements for tactile sensing devices. In particular, the complete coverage and the efficient coupling to the robot surface [85,86] can actually be accomplished with a continuous sensing layer covering the machine surface. This requirement is however difficult to achieve when using MEMS piezoresistors, which in contrast show a localized and punctual characteristic.

In contrast, high resolution and precision for the robot interaction with the external environment can be easily achieved by a distributed array of tactile sensors throughout the whole surface using a composite piezoresistive material. However, one of the major disadvantages of the piezoresistive composite based on the percolative working mechanism is the low dynamic range of sensitivity. This problem can be successfully overcome using piezoresistive composite materials based on quantum tunnelling conduction mechanism.

In these composites a small variation of the external load induces a huge change of the electrical conductivity [64,87,88], and thus an increase of the sensitivity. The conductive particles are dispersed very close to each other, however they remain fully coated with a polymeric layer because of their particular morphology. Therefore, the main difference with respect to the percolative composites consists in the permanent separation of the particle from each other by a thin layer of insulating polymer, representing the tunnelling barrier [89]. In this composite, even if the content of filler is well above the expected percolation threshold, no percolation path are formed in the uncompressed state and also during the application of a compressive forces in the pressure range of interest of tactile sensor.

**Figure 13.**
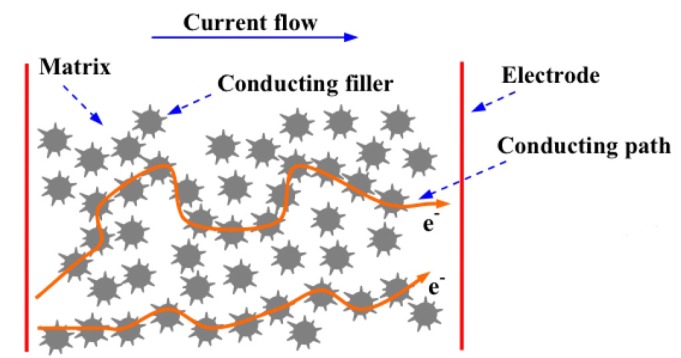
Scheme of the quantum tunnelling conduction mechanism in insulator-to-conductor piezoresistive composite under uniaxial pressure.

The quantum tunnelling mechanism is achieved thanks to the particular morphology of the conductive fillers, presenting either sharp and nanostructured tips at the surface or very high aspect ratios of the particles. In general, in the absence of any mechanical deformation, the resistance value of the whole composite is extremely high (it is comparable to the matrix one), leading to an insulator. In contrast, when the material is compressed, stretched or twisted, the mechanical deformation induces a reduction of the polymer layer thickness among the conductive spiky fillers. Therefore the tunnelling barrier decreases and the fillers form a sequence of tunnelling pathways. The probability of tunnelling phenomena increases, leading to a large reduction of the overall electrical resistance of the composite, as sketched in Figure 13.

The shape and dimension of the conductive filler particles play therefore a fundamental role in the quantum tunnelling mechanism, as well as the filler nature and amount [90]. In particular, the sharp and nanostructured tips at the filler surface are responsible for a local electric field enhancement [91] which considerably increases the probability of tunnelling through the polymeric insulating barrier [64,92].

The major drawback of these composites, as for the percolative ones, is the low repeatability of the measurements. Since the matrix is normally composed of an elastomer, the sensing materials present hysteresis phenomena when cycling measurements are performed. In contrast no destruction of the sharp tips on the particles surface occurs in the tactile pressure range because the soft matrix prevents any damage.

During the last decade, different models were used to explain the conduction mechanism, such as electrical field induced emission [93], Richardson-Schottky transmission types and Pole-Frenkel conduction [94]. However all these models only represent secondary order conduction and are therefore negligible mechanisms. In contrast, the tunnelling conduction is the dominant mechanism in these composites, whereas the percolation one can be considered negligible in the pressure ranges conventionally used to test the tactile sensor devices. However, it has to be noted that for very high pressures the percolation becomes predominant because the contact between conductive particles strictly close to each other cannot be avoided, even in the presence of the thin insulating polymeric layer.

In piezoresistive composites the whole electrical resistance is a function of both the resistance through each conducting particle and the polymer matrix. Assuming that the resistance of the matrix is constant everywhere, the resistance of the paths perpendicular to the current flow may be neglected, and, thus, the number of conducting particles between electrodes becomes a factor in this relationship, as well as the number of conducting paths [59]. The total resistance can then be described as:

(8) $$R=\frac{(L-1)R_{m}+LR_{c}}{S}\approx \frac{L(R_{m}+LR_{c})}{S}$$

where *R* is the composite resistance, *R_m_* the resistance between two adjacent particles, *R_c_* the resistance across one particle, *L* the number of particles forming one conducting path, and *S* the number of conducting paths.

From Equation (8) it is clear that the overall electrical resistance *R* in polymer composites is the resistance between two neighboring particles (*R_m_*) within the conducting network. *R_m_* is in turn dictated by the quantum mechanical tunnelling through the insulating polymer layer [95] and therefore is a function of the interparticle separation.

Starting from this simple Equation (8), the experimentally observed piezoresistance in quantum tunnelling composite is found to be well described by mathematical models based on quantum mechanical tunnelling mechanism [59,60,65,96], as described in the following.

The basic unit in these piezoresistive mathematical models is constituted by the tunnelling junction composed by two particles, separated by an insulating polymer layer. The computation is then extended to the whole sample, by considering the conductive paths across the material as constituted by chains of tunnelling junctions. The tunnelling current flowing at low applied voltage in the basic unit of the model was expressed by Simmons [97] as:

(9) $$I=\frac{3a^{2}\sqrt{2m\varphi }}{2d}{\left(\frac{e}{h}\right)}^{2}V\exp\left(-4\pi d\frac{\sqrt{2m\varphi }}{h^{2}}\right)$$

where *m* and *e* are the electron mass and charge respectively, *h* the Plank's constant, *V* the applied voltage, *d* and *φ* are the width and the height, respectively, of the potential barrier between two adjacent particles and *a^2^* is the effective cross-sectional area where the tunnelling occurs.

Starting from this equation, Zhang *et al.* [59,96] compute the resistance of a single barrier and then, considering the effect of all the tunnelling paths, the total resistance *R* of the composite sample as:

(10) $$R=\frac{L}{S}\left(\frac{4\pi hd}{3a^{2}\gamma e^{2}}\exp(4\pi \sqrt{\frac{2m\varphi }{h^{2}}}d)\right)$$

where *L* is the number of particles forming one tunnelling path and *S* is the total number of paths in a sample.

Assuming that the application of a uniaxial pressure would induce a reduction of the interparticle separation and thus an increase of the tunnelling probability, the piezoresistive variation in these quantum tunnelling composites can be expressed as:

(11) $$R=R_{0}(1-\frac{p}{G})\exp\left(-4\pi \sqrt{\frac{2m\varphi }{h^{2}}}d_{0}\frac{p}{G}\right)$$

where *p* is the applied pressure and *G* the composite compressive modulus.

Interestingly the piezoresistive composites based on the tunnelling conduction mechanism also show huge variations of electrical resistance when subjected to tensile pressure. It is usually expected that stretching the material would lead to an increase of the interparticle gap in the direction of the force, thus increasing the electrical resistance. However, since the polymeric matrices used in these composites are commonly nearly or purely incompressible, when stretched in one direction the material contracts in the directions perpendicular to the applied load by keeping the volume constant. This effect would cause the particles to get closer to each other in the direction perpendicular to the applied force. Since the conductive particles are randomly distributed along the material and are not perfectly aligned along planes, the deformation in the direction perpendicular to the stretching would produce a redistribution of the fillers in the composite, thus creating tunnelling paths along the samples. In this way, the resistance of the sample decreases exponentially with the applied pressure by following the tunnelling conduction mechanism and this variation can be expressed as follows [96]:

(12) $$R=\frac{R_{0}}{2\sqrt{1+\frac{p}{G}}}\text{exp}\left(-2\gamma \frac{\sqrt{1+\frac{p}{G}}-1}{\sqrt{1+\frac{p}{G}}}\right)$$

The first material exploiting the tunnelling conduction mechanism was presented by Bloor *et al.* in 2005 [64,98] by mixing nickel particles in an elastomeric matrix, and subsequently a tactile device fabricated with QTC was implemented on the NASA Robonaut [99]. Their material shows an electrical resistance reduction up to twelve orders of magnitude when compressed, stretched or bended.

Several scientific works [63,64,82,98,100] have also reported on the use of different metal micro- and nano-particles showing nanostructured spiky tips as fillers in tunnelling conduction composites. In particular, the shape and dimension of the filler particles was demonstrated to be very relevant on the final piezoresistive performances of the composite [90]. In that work a comparison between three different metal fillers in PDMS-based composites is reported: commercial nickel [100] and copper [42,101] spiky-particles, and chemically synthesized highly pointed gold nanostars [87].

The composites were in general prepared by incorporating the metallic powders in the PDMS by gentle mixing, in order to avoid the destruction of the tips on the surface of the particles. In the absence of an applied pressure, the electrical resistance of the nickel and copper composite was circa 1 GΩ, while for the gold composite was around 100 GΩ.

**Figure 14.**
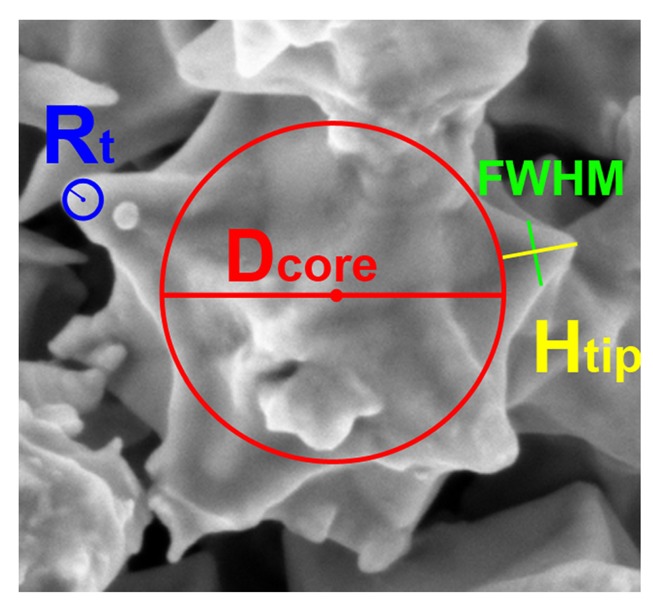
The scheme of the geometrical parameters considered in the comparison study of quantum tunnelling piezoresistive composites. Reprinted from [90].

Different figures of merit concerning the morphology of the fillers were evaluated and correlated with the corresponding functional response of the composites. In particular the authors took in consideration the following morphological parameters: (i) the average tip radius *R_t_*, (ii) the aspect ratio between the height (*H_t_*) and Full Width at Half Maximum (*FWHM*) of the tip, and (iii) the ratio between the *H_t_* and the particle core diameter (*D_core_*). Figure 14 shows a scheme of these morphological features.

The obtained results showed how the morphological features of the nanostructured particles can influence the minimum and the maximum required amount of the fillers to obtain similar piezoresistive performances among the different composites. This allowed therefore the selection of the best filler and easy tuning of the functional properties of the composites in order to reach the required sensor sensitivity.

**Figure 15.**
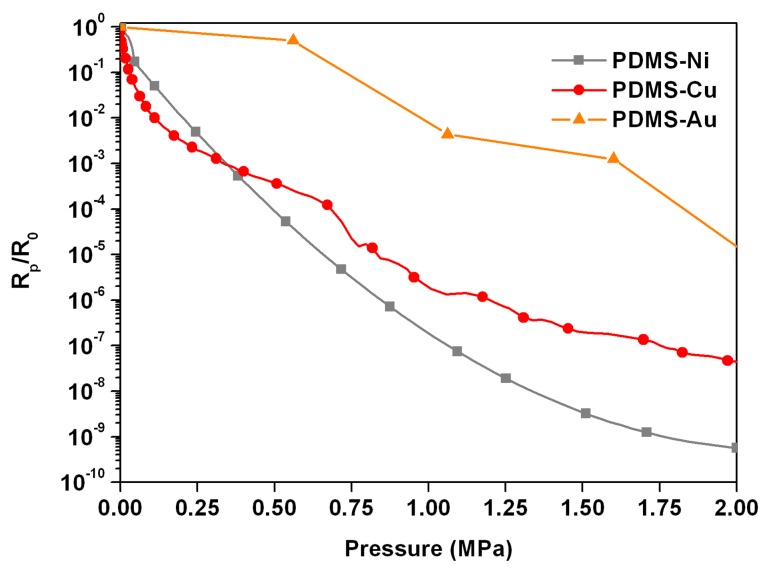
Electric resistance variation of the piezoresistive composites, obtained with the minimum filler amount, as a function of the applied uniaxial pressure.

The three composite samples were characterized under compressive pressure up to 2 MPa. The measured electric resistance variations of the three composites are plotted in Figure 15 as a function of the applied mechanical pressure, obtained for the optimized compositions of the final composites. In particular, from the experience acquired in their previous works [42,87,100], the authors were able to identify the minimum weight amount per each kind of filler required to obtain an appreciable and comparable tunnelling conduction effect between the different composites. Therefore, the weight ratio of 3:1 for the PDMS-Ni, 2:1 for PDMS-Cu, and 1:1 for the PDMS-Au composites were used [90]. Obviously, lower filler amounts with respect to the indicated ratios would have led to an insulating behaviour of the overall composites.

As explained above, here a negative pressure coefficient effect (NPCR) was observed: when subjected to mechanical compressive load, the polymer thickness separating each metal particle reduced, and the probability of tunnelling phenomena increased, resulting in an exponential reduction of the bulk electrical resistance. This effect is particularly evident for both PDMS-Ni (grey curve) and PDMS-Cu (red curve) composites. In particular, the gauge factors, calculated with the method of Abyaneh and Kulkarni [88], were about 18 for the nickel-based composite, and approximately 10 for both the PDMS-Cu and PDMS-Au ones. These gauge factors provide an evaluation of the strain sensitivity of the aforementioned composites, and these values could be enhanced by increasing the metallic filler to polymer ratio. However, one should note that the PDMS-Ni composite showed the highest filler to polymer ratio (3:1), whereas only a ratio 1:1 was employed for obtaining a piezoresistive response from the PDMS-Au composite. This difference can be clearly understood by analyzing the geometrical features of the three different metal particles, as reported in Table 7.

Both copper and gold particles have higher *H_tip_*/*D_core_* and *H_tip_*/*FMWH* ratios with respect to nickel ones. Both figures of merit imply that the tips were sharper and slender, thus being responsible for the local electric field enhancement that considerably increased the tunnelling probability through the polymeric insulating barrier in the composite. Both PDMS-Cu and PDMS-Au composites showed indeed a remarkable tunnelling conduction behaviour at lower filler amounts (2:1 for PDMS-Cu and 1:1 for PDMS-Au) with respect to the weight ratio used for the nickel-based composite (3:1).

In addition, thanks to their very small tip radius (*R_tip_*), the gold nanoparticles could give the better performances in term of lower filler to polymer ratio, producing similar values of piezoresistance. The advantages of using spiky and nanosized particles, like the gold one presented here and in the previous works [88,91], drastically reduces the piezoresistive film thickness and promote the integration with MEMS technologies.

Similarly, silver nanostructures were employed as conductive fillers for functional sensing composites. Hong *et al.* [102] investigated the electrical and thermal conductivities of a silver flake/thermosetting polymer composite. The silver flake size, the filler distribution and amount into the polymer matrix were studied as relevant parameters influencing the electrical volume resistivity and the thermal conductivity of the composite. Therefore, concerning the materials used for quantum tunnelling composites, the great advantage is to use nanometer sized and nanostructure-shaped fillers, in order to obtain flexible, thin, and light-weight performing piezoresistive composites, which can be well adaptable to tactile sensor applications.

A part these issues, the research has also to deal with the tactile sensor positioning and architecture on the robot surface, the electronic configuration and hardware, the methods to access and acquire the sensing data, and the algorithms to process and interpret the acquired signal in real time [4,103].

Generally speaking, when the tactile skin consists in a continuous layer of functional composite sandwiched between a matrix of top and bottom electrodes, one already has an array of distributed and numerous tactile sensors over the robot surface. A high density of sensors can generate sufficient data to improve the precision of the robotic motion in an unstructured environment and object recognition. However, the large density of sensors and their spatial positioning have to be accurately matched with the current technology for data handling and processing. Therefore this issue can be a limitation to the continue miniaturization and high density of distributed tactile sensors. A continuous improvement in the increase of maximum essential data extraction is mandatory for an efficient tactile signal processing.

Design of an *ad-hoc* electronics able to read out the sensing signals and further process them to obtain data representation and an as much as possible realistic interpretation was recently developed [43]. In that work, the authors integrated a continuous and flexible piezoresistive quantum tunnelling composite based on spiky nickel particles with a customized electronic read-out circuit. The circuit was able to read the resistance variations of the composite sensor upon a compressive load and process these signals upon their spatial positioning with a software interface. As a result, a real-time tridimensional graphical representation of the compressed region on the tactile device was achieved.

In details, the developed sensor was prepared by the hot embossing technique (area: 40 mm × 40 mm and thickness: 1 mm) with an 8 × 8 electrode matrix obtained by patterning two copper metalized polyimide foils used as bottom and top electrodes, respectively. For the data acquisition, the distributed sensing area was modeled as a two-dimensional array of resistor nodes, which resistance varied upon the exerted pressure intensity on each node. The instantaneous resistance value was obtained by measuring the current flowing through each node at a fixed voltage. Each resistor of the matrix was connected to the measuring circuit through two analog multiplexers used for monitoring rows and columns respectively. The presented quantum tunnelling piezoresistive device was able to vary each node resistance by several decades, up to nine orders of magnitude. This huge sensitivity can be a problem for data acquisition by the read-out circuit. The challenge was successfully overcome by using a single temperature compensated logarithmic transimpedance amplifier, ensuring an almost uniform profile of the current, with respect to the huge and exponential variation of the current versus the applied pressure in each node.

**Figure 16.**
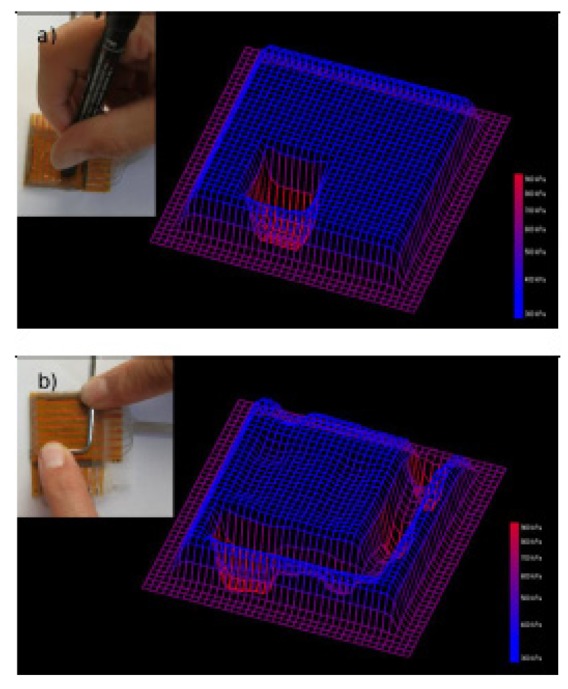
3-D visual representation of the distributed pressures on the tactile sensing matrix when a load is applied on selected nodes: (**a**) a punctual compressive strain applied with a pen, and (**b**) a manually pressed hex key. In blue are represented the uncompressed nodes, whereas in red are the nodes which dynamic saturate under the applied compressive strain. Reprinted from [43], Copyright (2013), with permission from Elsevier.

In order to obtain a real-time response and further visualization of the sensing area, the measurements on each matrix node were carried out at a frequency of 1 kHz; therefore the whole 8 × 8 matrix was sampled at a frequency of about 16 Hz. A microcontroller performed a continuous and sequential scanning of the nodes and sent the data to a PC. After the completion of 64 measurements, the PC software plotted a grid where each node corresponded to the sensor matrix node. In Figure 16 the height of each grid node and thus the color scale in the 3-D representation corresponded to the pressure applied on each corresponding site on the tactile sensor. To improve the visual 3-D representation with smooth transitions among each node, the represented grid was increased up to 34 × 34 nodes [43].

**Figure 17.**
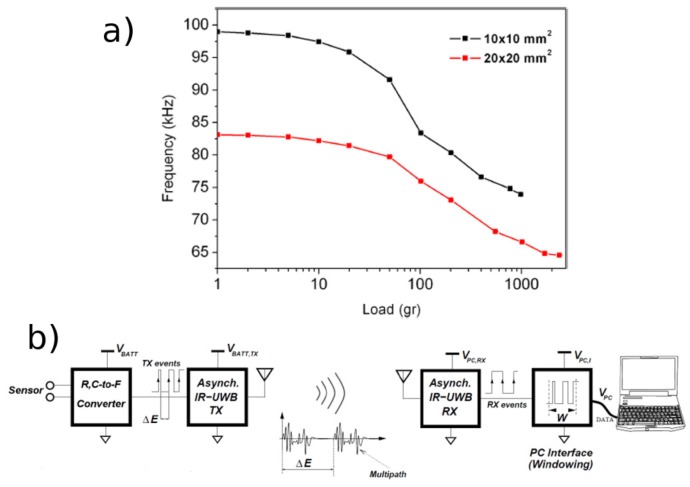
(**a**) Resonant oscillating frequency as function of the applied static load for two different sensors and (**b**) schematic of the whole sensing device.

Recently it was also proposed a completely innovative approach to measure the applied load on the quantum tunnelling piezoresistive composites reported above, by exploiting not only their electrical resistance variation under the application of a pressure, but also their capacitance was also proposed [44]. Since the material basic unit consists of an insulating dielectric layer (the tunnelling barrier) between two metallic particles, it forms a capacitor. When deformed, the interparticle layer decreased, increasing the capacitance of the unit and consequently the one of the whole composite sample. A new sensor architecture was designed to exploit both the resistance and the capacitance variation to measure pressure with a very high sensitivity and fast response [44]. The read-out circuit exploits a quasi-digital solution, converting resistance (*R*) and capacitance (*C*) values of the sensor in to a frequency (*F*) signal. The *R*, *C* to *F* converter guarantees low power consumption, complexity and dimension being completely fabricated with CMOS technology [104]. The signal is then wireless transmitted to the PC interface for elaboration by an Impulse-radio Ultra-wide-band (IR-UWB) transmitter [105]. The quasi-digital signal is compatible with the IR-UWB circuit and can be transmitted without any further elaboration, reducing transmission time and power. The signal is then elaborated by a PC which converts frequency values into pressure ones. A schematic of the system is presented in Figure 17b. This solution can be really interesting for implementation on robots, because thanks to the small dimensions of the circuit it is possible to increase the number of converters in a single CMOS chip, controlling several sensors simultaneously without using a multiplexer, which reduces the read-out process. Moreover the wireless transmission can reduce the cable complexity on the robotic machine.

In this configuration the piezoresistive sensor is extremely sensitive [45]. Static pressure measurements were performed on the sensing element by applying different loads. Thanks to this measurement system it was possible to resolve 1 g of applied load, as reported in Figure 17a. The system is very sensitive to small load, while it tends to saturate for higher ones (over 1,500 g). Samples with different area (10 × 10 mm^2^ and 20 × 20 mm^2^) were tested, showing different variations of the resonant oscillation frequency and demonstrating the very high sensitivity of this type of sensor.

## 6. Conclusions

The analysis of the state-of-the-art of tactile sensor devices evidences a predominance of solutions exploiting piezoresistivity as transduction mechanism. The ease and highly reproducible fabrication techniques have shown to be fundamental for the diffusion of this kind of devices. However, the key parameter is the wide range of functional materials available for the sensor development. The choice of the sensing material allows indeed tuning the working range of the sensor, reaching high sensitivity and obtaining different mechanical properties and robustness, depending on the application and environment of utilization.

Among the devices based on resistive solutions, flexible composite materials give the possibility to satisfy quite all the requirements presented in Table 1, because of the quite infinite combination of properties obtainable by mixing different materials, varying composition ratio and orientation of the filler. The main limitation of the piezoresistive materials is still represented by the hysteresis in the sensor output that could reduce the sensitivity and the repeatability. However several solutions to overcome this problem were investigated in the choice of the materials as well as in the conditioning electronics, leading to some works with performing and repeatable results.

Here, the review of the tactile sensors studies exploiting flexible piezoresistive composites is divided according to the conduction mechanism in piezoresistors, strain gauges, percolation and quantum tunnelling devices. Alternative classifications could have also be done based on the type of functional materials, system architectures or mechanical properties; however the selected organization allowed also a differentiation of the presented sensors corresponding to their sensitivity and applications. The majority of the reviewed solutions are based on classical read-out architecture for resistive sensors, even if some innovative techniques are presented, such as organic field emitter transistor (OFET) and resistive and capacitance to frequency (*R*, *C* to *F*) converter, resulting in high sensitive devices.

Tactile devices with piezoresistive composites as sensing materials were already reached, even with some limitations. Improved performances can thus be thus obtained, allowing an effective utilization in robots and returning reliable and rapid pressure feedback signals for performing exploration, dexterous manipulation and control tasks.

Moreover a new trend in developing multifunctional devices is growing faster. New perspective solutions measuring on the same chip either pressure (also in the three dimensions), temperature, hardness and thermal conductivity were recently developed and constitute the new field to which the tactile sensors can expand and feed new research perspectives.

## Figures and Tables

**Table 1.** Specific requirements for the design of tactile sensor devices to be implemented on human robots. Adapted from [3], Copyright (2011), with permission from Elsevier.

**Parameter**	**Requirements**
Force direction	Both normal and tangential
Force range	0.01 N–10 N (1000:1)
Temporal variation	Both dynamic and static
Time response	1 ms for tactel (depending on the dimension for arrays)
Spatial resolution	1 mm at fingertips, 5 mm on palm, even less on limbs and belly
Sensor output	Stable, repeatable, monotonic and low hysteresis
Array output	Minimal or null cross-talk
Sensing Surface	Compliant and durable
Mechanical properties	Flexible, conformable, stretchable and robust (depending on the application and environment)
Shielding	Electronic and magnetic shielding
Data organization	Preprocessing to reduce data to central unit
Fabrication	Simple mechanical integration, minimal wiring, low cost
Electronics	Low power consumption

**Table 2.** Comparison of the different flexible composite tactile sensors.

**Parameter**	**Sensor Type**			

	**Piezoresistors**	**Strain Gauges**	**Percolation Mechanism**	**Quantum Tunnelling Mechanism**
Sensitivity	High sensitivity	High sensitivity	Low sensitivity	High sensitivity
Repeatability	High repeatability	High repeatability	Hysteresis Problem	Hysteresis Problem
Spatial resolution	High	Quite high	Low (except OFET)	Low
Working area	Suitable for small area (*i.e.*, fingertip)	Suitable for small area (*i.e.*, fingertip)	Small and large area	Small and large area
Working range	Low	Medium	Low, but tunable with composition	Very high and tunable
Fabrication techniques	Costly materials and techniques	Costly materials and techniques	Simple fabrication techniques	Simple fabrication techniques
MEMS/electronic integration	Ease integration	Ease integration	Complex integration (except OFET)	Complex integration
Mechanical properties	Fragile (better with protective elastomer)	Fragile (better with protective elastomer)	Stretchable and robust	Stretchable and robust

**Table 3.** Comparison of tactile sensor solutions based on flexible piezoresistor.

**Year**	**Author**	**Functional Material**	**No. of Sensing Elements**	**Spatial Resolution**	**Signal Conditioning Circuit**	**Working Range**	**Sensitivity**
2013	Ahmed *et al.* [22]	Nichrome	48	283 μm	Yes	0–30 kPa	1.25 V/N (average)
2013	Koiva *et al.* [23]	Metal	12	5.5 mm	Yes	0–10 N	-
2006	Noda *et al.* [24]	Silicon	1	20 mm	No	0–5 kPa (−5 kPa to 5 kPa shear)	0.015% (0.03% shear)
2008	Beccai *et al.* [25]	Silicon	1	3 mm	Partially	0–15 N (0–11 shear)	100 mV/N (400 mV/N shear)

**Table 4.** Comparison of tactile sensor solutions based on flexible strain gauges.

**Year**	**Author**	**Functional Material**	**No. of Sensing Elements**	**Spatial Resolution**	**Signal Conditioning Circuit**		**Working Range**	**Sensitivity**	
2005	Engel *et al.* [26]	Nichrome	5 × 5	5 mm	Yes		-	340 ppm/mN	
2009	Kim *et al.* [27]	Nichrome	32 × 32	1 mm	Yes		0–1 N	2%/N (0–0.6 N) 1%/N(0.6–1 N)	
2010	Zang *et al.* [28]	Copper	-	400 μm	No		0–7 N	0.3%	
2010	Choi *et al.* [29]	Nichrome	4 × 4	2.5 mm	Partially		0–0.8 N	206.6 mV/N (70.1mV/N shear)	
2013	Tata *et al.* [30]	Carbon	1	20 × 5 mm	yes		0%–50% (*ε*)	24.15 mV/*ε* (%)	
2012	Lu *et al.* [31]	Carbon black-PDMS	10	-		No	-		4 mV/*ε* (%)

**Table 5.** Comparison of tactile sensor solutions based on percolation mechanism.

**Year**	**Author**	**Functional Material**	**No. of Sensing Elements**	**Spatial Resolution**	**Signal Conditioning Circuit**	**Working range**	**Sensitivity**
2011	Hwang *et al.* [32]	PDMS-CNTs	1	5 mm	No	0.0.12 MPa	-
2013	Pyo *et al.* [33]	PDMS-CNTs	1	-	No	0–2 N	∼5%–6%/N
2010	Lay *et al.* [34]	PDMS-CNTs	8 × 8	1.5 mm	Partially	0–9 N	0.145%/mN
2010	Yang *et al.* [35,36]	Press.-cond. Rubber a	32 × 32	3 mm	Yes	0–650 kPa	-
2000	Yuji *et al.* [37]	Press.-cond. Rubber a	1	30 mm	Yes	0.2–1.6 kgf	-
2011	Cheng *et al.* [38,39]	Conductive polymer b	8 × 8	3 mm	Yes	0–650 kPa	300 Ω/kPa
2004	Shimojo *et al.* [40]	Press.-cond. Rubber a	16 × 3	3 mm	Yes	0–0.2 MPa	-
2005	Somaya *et al.* [41]	Press.-cond. Rubber a	12 × 12 (OFET)	4 mm	Yes	0–30 kPa	0.2 μA/kPa

a Pressure-Conductive Rubber (carbon particles in elastomer matrix);

b PDMS filled with carbon black, copper and silver particles.

**Table 6.** Comparison of tactile sensor solutions based on quantum tunnelling mechanism.

**Year**	**Author**	**Functional Material**	**No. of Sensing Elements**	**Spatial Resolution**	**Signal Conditioning Circuit**	**Working Range**	**Sensitivity**
2011	Bloor *et al.* [32]	Elastomer-nickel	-	-	No	0%–25% strain	∼0.5 decade/%
2013	Stassi *et al.* [42]	PDMS-copper	8 × 8	2 mm	Yes	0–2 MPa	variable a
2010	Canavese *et al.* [43]	PDMS-nickel	8 × 8	1.5 mm	Yes	300–1,150 kPa	exponential
2010	Stassi *et al.* [44,45]	PDMS-nickel	1	10 mm	Yes	0–100 kPa	∼1.7 kHz/kPa (up to 10 kPa)

a 8.77 V/MPa (0–0.25 MPa), 2.21 V/MPa (0.25–0.85 MPa), 0.63 V/MPa (0.85–2 MPa).

**Table 7.** Geometrical parameters of the nanostrucutred spiky particles. Reprinted from [90].

**Metal Particles**	**R_t_^a^ [nm]**	**H_tip_/FWHM b**	**H_tip_/D_core_^c^**	**Filler:PDMS Weight Ratio**
Ni	43	1.1	0.09	3.1
Cu	975	3.6	0.37	2:1
Au	17	2.3	0.34	1:1

a Average tip radius;

b Aspect ratio between the tip height (H_t_) and its full-width at half-maximum (FWHM);

c Ratio between the tip height (H_t_) and the core diameter of the particle (D_core_).
